# Human islets contain four distinct subtypes of β cells

**DOI:** 10.1038/ncomms11756

**Published:** 2016-07-11

**Authors:** Craig Dorrell, Jonathan Schug, Pamela S. Canaday, Holger A. Russ, Branden D. Tarlow, Maria T. Grompe, Tamara Horton, Matthias Hebrok, Philip R. Streeter, Klaus H. Kaestner, Markus Grompe

**Affiliations:** 1 Oregon Stem Cell Center, Papé Family Pediatric Research Institute, Department of Pediatrics, Oregon Health and Science University, 3181 SW Sam Jackson Park Road, Portland, Oregon 97239, USA; 2 Department of Genetics and Institute for Diabetes, Obesity, and Metabolism; University of Pennsylvania School of Medicine, Philadelphia, Pennsylvania 19104, USA; 3 Diabetes Center, Department of Medicine, University of California San Francisco, San Francisco, California 94143, USA

## Abstract

Human pancreatic islets of Langerhans contain five distinct endocrine cell types, each producing a characteristic hormone. The dysfunction or loss of the insulin-producing β cells causes diabetes mellitus, a disease that harms millions. Until now, β cells were generally regarded as a single, homogenous cell population. Here we identify four antigenically distinct subtypes of human β cells, which we refer to as β1–4, and which are distinguished by differential expression of ST8SIA1 and CD9. These subpopulations are always present in normal adult islets and have diverse gene expression profiles and distinct basal and glucose-stimulated insulin secretion. Importantly, the β cell subtype distribution is profoundly altered in type 2 diabetes. These data suggest that this antigenically defined β cell heterogeneity is functionally and likely medically relevant.

## Abstract (web summary)

Dysfunction or loss of insulin-secreting β cells in the pancreas is a hallmark of diabetes. Here, Dorrell *et al.* identify four subpopulations of β cells in humans, which differ in gene expression and insulin secretion kinetics, and the abundance of which is altered in patients with type 2 diabetes.

## Graphical abstract

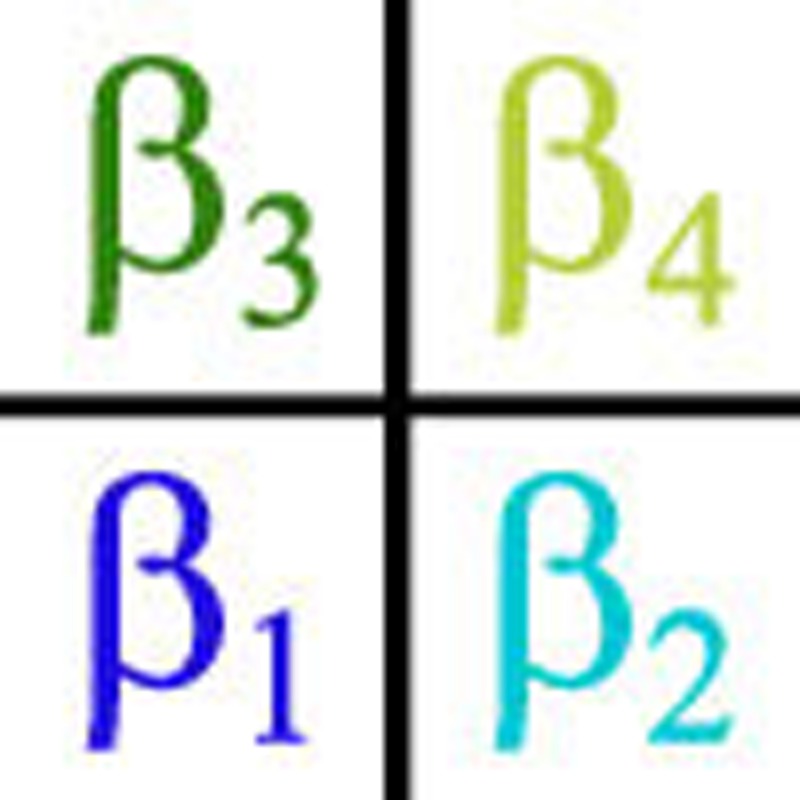

Pancreatic β cells are the glucose-responsive, insulin-secreting metabolic sensor population of the Islets of Langerhans^1^. Although the cellular composition of islets is heterogeneous, including α, β, δ, γ/PP and ɛ endocrine cells and supporting vasculature, β cells have been thought to be a homogeneous cell type. Despite this prevailing paradigm, there have long been hints of functional heterogeneity^2^,^3^. *In vitro* studies of individual rat β cells have revealed variable glucose responsiveness and insulin secretion upon challenge^4^. Rat studies also provided evidence of marker heterogeneity; both a polysialylated form of neural cell adhesion molecule (PSA-NCAM)^5^ and CDH1^6^ were shown to be overrepresented on β cells with high insulin secretion capacity. In human islets, SLC18A2/VMAT2 (ref. 7) and DKK3 (ref. 8) were found in β cell subsets and heterogeneity in insulin secretion has also been suggested^9^. In this report, we explore human β cell heterogeneity with new markers and identify subpopulations present at different frequencies in healthy and type 2 diabetes (T2D) islets. Dissimilar basal and glucose-stimulated insulin secretion (GSIS) characteristics indicate that these subtypes are functionally distinct, and suggest possible clinical relevance.

## Results

### Development of antibodies to assess cellular heterogeneity

To study cell type heterogeneity in the human pancreas, we developed cell surface marking antibodies by immunizing mice with human islets. These monoclonal antibodies permit the labelling, isolation and study of ducts, acinar cells and endocrine cells^10^,^11^. Live human pancreatic β cells were purified with fluorescence-activated cell sorting (FACS) using the combination of positive selection with the pan-endocrine marker HPi2 (HIC1-2B4) and negative selection for HPa3 (HIC3-2D12), an antibody that labels all non-β endocrine cell types (Supplementary Fig. 1)^11^. To determine whether this ‘pure' HPi2^+^/HPa3^−^ β cell population was actually heterogeneous, we examined numerous cell surface antigens known to be expressed on β cells from transcriptome analysis^10^ and systematically analysed our novel monoclonal antibody collection^12^ for subset binding. Two antibodies exhibited clear antigenic heterogeneity within the β cell compartment: HIC0-3C5, a novel monoclonal antibody developed in our anti-islet screens and monoclonal antibodies recognizing CD9, a tetraspanin identified as a β cell marker in our transcriptome analyses. To identify the antigen for HIC0-3C5, rat C6 cells carrying a human cDNA library were screened by FACS isolation and the re-growth of positively labelled cells (Supplementary Fig. 2). The HIC0-3C5 antigen was revealed by cDNA insert sequencing to be ST8SIA1, an alpha-N-acetylneuraminide alpha-2,8-sialyltransferase of unknown function in endocrine cells^13^. Neither of these β cell-subset markers were expressed exclusively in this cell type; in the pancreas, ST8SIA1 is found on about half of α cells and CD9 is present on most δ cells (Supplementary Fig. 3a,b).

### β cells can be subdivided into antigenic subtypes

**Figure 1:**
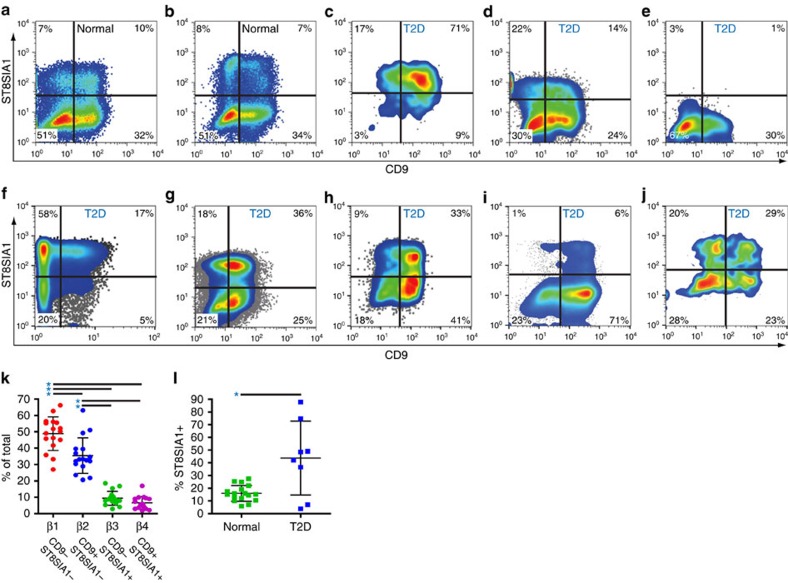
β cells are antigenically heterogeneous in normal and pathological islets. Human islet samples were enzymatically dispersed and antibody labelled for flow cytometric analysis, and β cells were isolated by FACS with the sorting scheme illustrated in Supplementary Fig. 1. (**a**,**b**) Representative examples of CD9 versus ST8SIA1 expression on the purified β cell populations from two healthy islet donors. (**c**–**j**) CD9 versus ST8SIA1 expression on cells from eight islet samples collected from T2D patients. (**k**) Mean β cell subtype distribution (±s.d.) from 17 normal islet preps. Significant frequency variance (*P*<1 × 10^−24^, single factor analysis of variance) was observed between these populations and β1 is significantly more frequent (*P*=8 × 10^−4^, 1.2 × 10^−12^, 5 × 10^−13^ by *t*-test (unequal variance)) than any of the others and β2 was significantly more frequent than β3 or β4 (1 × 10^−8^, 2 × 10^−9^ by *t*-test (unequal variance)) as indicated with asterisks. (**l**) The mean percentage (±s.d.) of ST8SIA1^+^ β cells is abnormally high (*P*=0.028, *t*-test (unequal variance)) in T2D islets (*n*=8) compared with normal islets (*n*=17).

**Figure 2:**
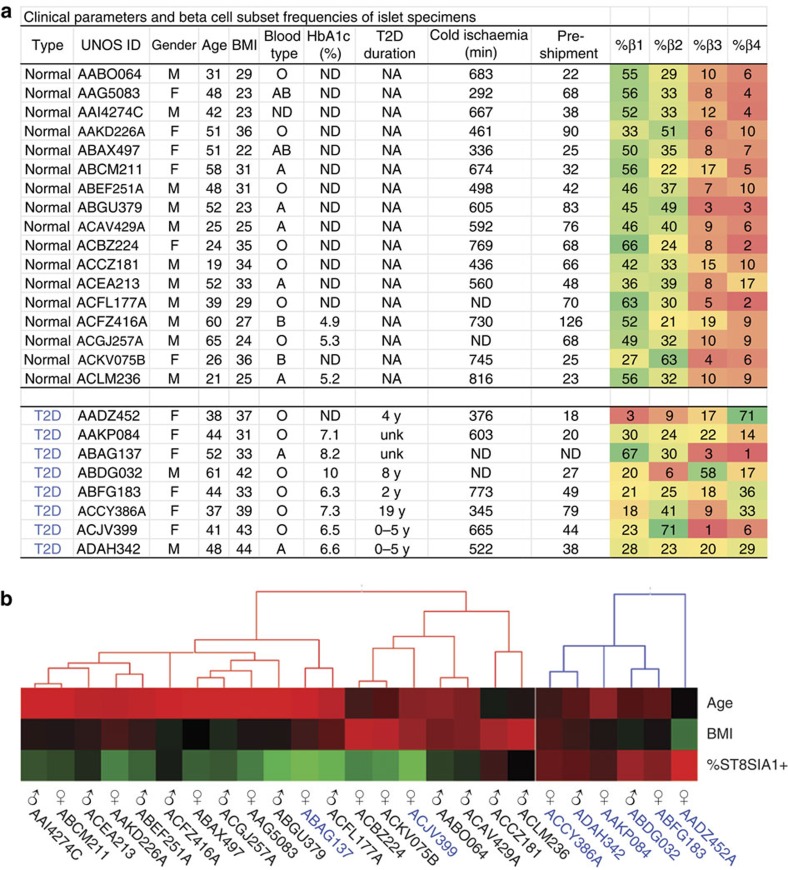
Clinical parameters and subset frequencies of human islet specimens. (**a**) Donor information corresponding to islets obtained from 17 healthy and 8 diabetic donors. HbA1c, glycated haemoglobin; T2D, type 2 diabetes. Duration of cold ischaemia refers to the time of pancreatic cold storage in University of Wisconsin solution before islet isolation. (**b**) Unsupervised clustering of a subset of clinical parameters and the percentages of ST8SIA1^+^ β cells measured by FACS were visualized using Hierarchical Clustering Explorer 3.5.

Live pancreatic β cells were isolated from human islet samples by FACS and co-labelled with antibodies recognizing ST8SIA1 and CD9, revealing four antigenically distinct subpopulations (Fig. 1). We labelled these β1–4, with β1 being most abundant and β4 most rare. Transmission electron microscopic imaging of evaluable populations revealed comparable structures and confirmed the presence of insulin granules in each subtype (Supplementary Fig. 4). The subtype frequencies were similar in 17 healthy individuals (Fig. 1k), and all four subpopulations were present in each case. β1 was the largest subpopulation, followed by β2 and the minor β3 and β4 populations. Subset frequencies were compared with available clinical parameters including gender, age, body mass index and time of cold ischaemia for the donor and specimen, but no significant correlations were observed. Importantly, the subtype percentages of healthy obese individuals (body mass index>30) did not differ from healthy, lean people (Fig. 2).

### Assessment of β cell-subset markers in intact tissue

**Figure 3:**
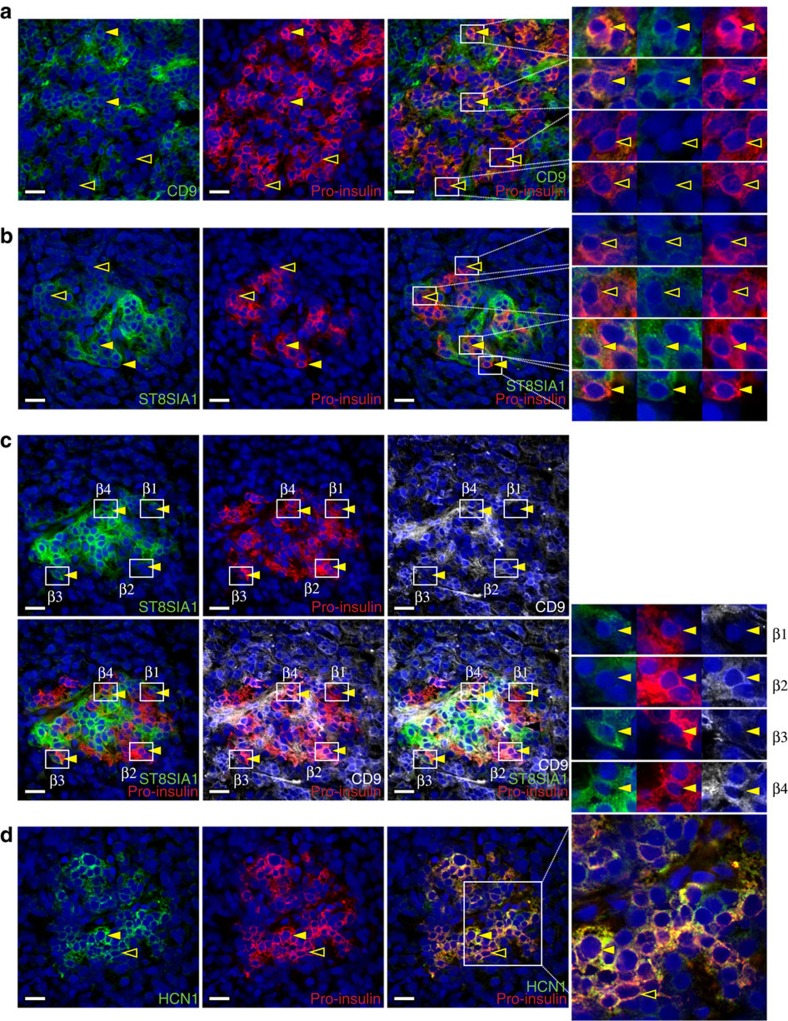
β cell heterogeneity in normal pancreatic tissue sections. (**a**) Human pancreas co-labelled with antibodies recognizing CD9 and proinsulin. Most proinsulin^+^ β cells were CD9^−^ (hollow arrows) but a subset was CD9^+^ (solid arrows). (**b**) Tissue co-labelled with antibodies recognizing ST8SIA1 and proinsulin. Most proinsulin^+^ β cells were ST8SIA1^−^ (hollow arrows) but a subset was ST8SIA1^+^ (solid arrows). (**c**) Compound labelling of proinsulin, CD9 and ST8SIA1 reveals proinsulin^+^ cells expressing every combination of the other markers; examples of β1 (CD9^−^ST8SIA1^−^), β2 (CD9^+^ST8SIA1^−^), β3 (CD9^−^ST8SIA1^+^) and β4 (CD9^+^ST8SIA1^+^) cells are indicated. Note that both CD9 and ST8SIA1 are found on proinsulin^−^ (non-β) cell types as well, as shown in Supplementary Fig. 1. (**d**) HCN1 was detected in a subset of proinsulin^+^ β cells; examples of high/positive and low/negative cells are indicated with solid and hollow arrows, respectively. Tissues illustrated are 5 μm cryosections of normal human pancreas. Scale bar, 25 μm.

Human islet preparations from cadaveric donors are typically cultured for days before becoming available for analysis^14^,^15^. Therefore, it was possible that the observed antigenic β cell heterogeneity was the product of *in vitro* de-differentiation and not representative of true *in vivo* heterogeneity. To address this possibility, we assessed the expression of ST8SIA1 and CD9 in sections of human pancreata. Figure 3a illustrates co-labelling of CD9 and proinsulin, the non-secreted precursor of (β cell-specific) insulin. Most proinsulin^+^ β cells were CD9^−^, but a large subpopulation of proinsulin^+^CD9^+^ cells was present, as predicted by FACS analysis. Similar heterogeneity, with a smaller proportion of positively marked β cells, was observed for ST8SIA1 (Fig. 3b). Quantification of the subtype frequencies in 100 tissue-resident islets fell within the range expected from the FACS data (Supplementary Fig. 5). Combination labelling with both β cell subset markers directly illustrates the antigenic heterogeneity of human β cells (Fig. 3c and Supplementary Fig. 3c). These results demonstrate β cell heterogeneity *in situ*, indicating that it is not an artefact of islet isolation or culture. Protein-level evidence of heterogeneity was also observed for HCN1 (potassium/sodium hyperpolarization-activated cyclic nucleotide-gated channel 1; Fig. 3d), which had been identified as a potential β cell-subset marker by RNA expression analysis (Table 1).

### β cell subsets have differences in gene expression

**Figure 4:**
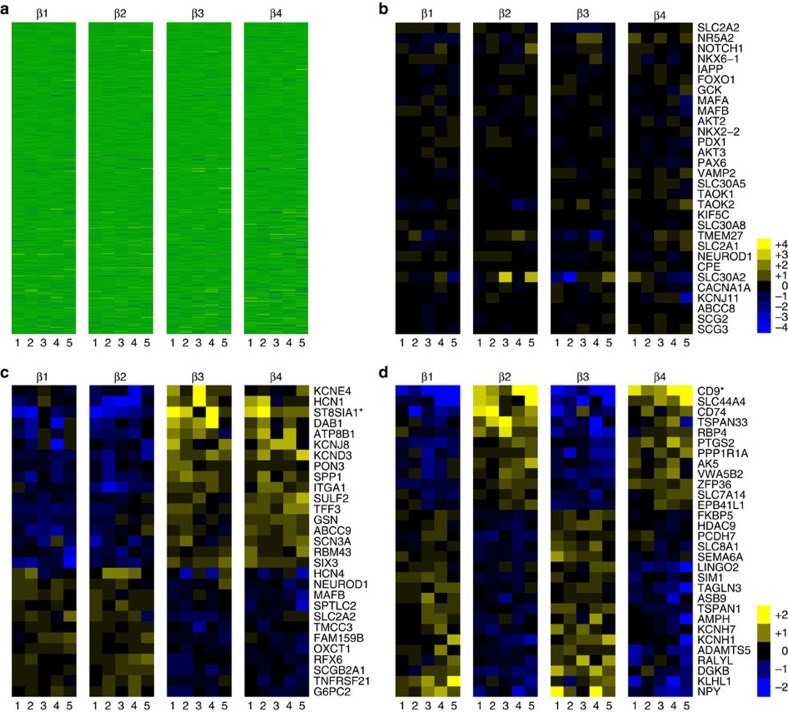
Comparative gene expression in β cell subtypes. RNA-seq data derived from β cells isolated from five different normal donors were normalized and compared with determine genes, which were differentially expressed. (**a**) All 23,292 transcripts for all samples arrayed as a heat map. (**b**) Known genes associated with insulin signalling/processing/secretion and β cell identity. (**c**) Genes differentially expressed between the ST8SIA1^+^ β3/β4 and ST8SIA1^−^ β1/β2 subtypes. (**d**) Genes differentially expressed between the CD9^+^ β2/β4 and CD9^−^ β1/β3 subtypes.

We next determined whether antigenic heterogeneity correlated with functional differences between the β cell subsets. Transcriptome analysis by RNA sequencing (RNA-seq) was performed on the β cell subsets of five healthy donors. Importantly, all four populations clearly displayed the gene expression profile of classic β cells with very high levels of insulin mRNA and other typical marker genes, such as *PDX1*, *MAFA* and *NKX6.1*. Indeed, the four populations are overall very similar in their RNA expression profile (Fig. 4a). A heat map showing the expression of a selected list of genes known to be important for β cell function is shown in Fig. 4b. Nonetheless, a subset of genes was consistently expressed at different levels in the β cell subtypes (Fig. 4c,d, Table 1 and Supplementary Figs 6–9). Using stringent statistical cutoffs, a sizeable list of genes (125 with false discovery rate≤0.1, 72≤0.05 and 49≤0.01) was found to be differentially expressed between the ST8SIA1-positive (β3/4) and -negative (β1/2) populations (Fig. 4c, Table 1 and Supplementary Figs 6 and 7) as well as the CD9^+^ and CD9^−^ populations (107 with false discovery rate≤0.1, 86≤0.05 and 45≤0.01, Fig. 4d and Supplementary Figs 8 and 9). Given the inter-individual heterogeneity inherent to human clinical specimens, these differences are highly significant and likely represent only a minimal estimate of the true transcriptome heterogeneity. Contamination with other endocrine cell types was negligible (Supplementary Fig. 10), but nonetheless all genes known to be robustly expressed in non-β pancreatic cells were removed from analysis. As expected, the genes encoding the antigens used for cell isolation, *CD9* and *ST8SIA1*, were expressed at significantly higher levels in the β2/4 and β3/4 populations, respectively (Fig. 4c,d, Table 1 and Supplementary Figs 6 and 8). Most of the differentially expressed genes are of unknown function in β cells, but some have been clearly associated with insulin secretion (for examples, *GLUT2* (ref. 16), *PPP1R1A*^17^ and *ABCC9/SUR2* (ref. 18)) or are known to be dysregulated in T2D (for examples, *G6PC2* (ref. 19, *RPB4* (ref. 20) and *MAFB*^21^). Interestingly, the ST8SIA1^−^ population is significantly enriched for GO biological processes related to protein secretion including ‘Regulation of Insulin Secretion' (*n*=9, *P*=0.012), whereas ST8SIA1^+^ is enriched for ‘Neurogenesis' (*n*=16, *P*=0.012). Thus, by gene expression, it might be expected that the ST8SIA1^−^ β cells are more capable of secreting insulin. Among the other genes expressed heterogeneously in β cells, one example with possible relevance to insulin secretion is the f-channel *HCN1*, which was increased threefold in the ST8SIA1^+^ (β3/4) populations. Co-labelling of HCN1 with proinsulin confirmed that β cells exhibit a range of HCN1 expression at the protein level (Fig. 3d). Several transcription factors were also differentially expressed, including *SIX3*, *RFX6*, *MAFB* and *NEUROD1.* Notably, *SIX3* was recently shown to be important for β cell maturation during ageing and higher expression levels were found to enhance insulin secretion^22^. Other previously reported human heterogeneity markers, that is, *VMAT2* (ref. 7) or *DKK3* (ref. 8) were not significantly differentially expressed in our subsets.

### Functional assessment of insulin secretion

**Figure 5:**
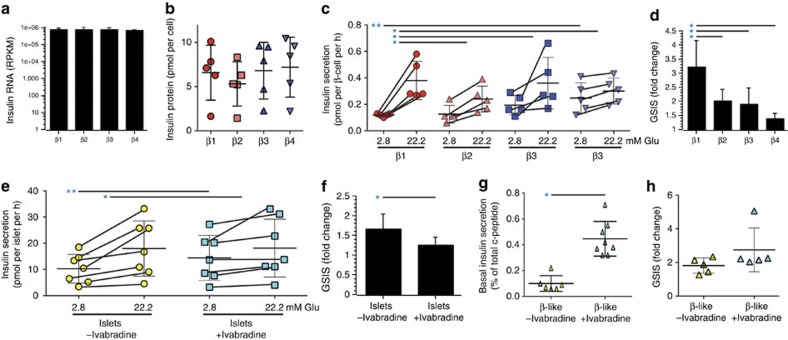
Functional characterization of β cell subtypes and islet cultures. No significant differences in the mean levels of insulin RNA (**a**) and insulin protein (**b**) were found in the four β cell subtypes (*n*=5 and *n*=3 specimens, respectively; all values reported as mean±s.d.). (**c**) Insulin secretion (pmol of insulin per β cell per hour) were measured by incubation of reaggregated cells under basal (2.8 mM glucose) and stimulating (22.2 mM glucose) conditions. Basal levels were significantly different only between β1 and β4 (*P*=0.037, *t*-test (equal variance)), but glucose-stimulated insulin secretion (GSIS) was significantly higher in the β1 subtype than in any of the other β cell subtypes (β1 versus β2: *P*=0.045, β1 versus β3: *P*=0.042, β1 versus β4: *P*=0.005, *t*-test (equal variance)). The significant basal secretion difference is indicated with a double-asterisk (**) and the significant GSIS differences are indicated with a single asterisk (*). (**d**) Fold-change representation of the GSIS values reported in **c**. (**e**) Insulin secretion (pmol of insulin per islet per hour) is shown for intact human islets exposed to basal and stimulating glucose concentrations with or without HCN inhibition by ivabradine (30 mM). Basal insulin secretion was significantly increased (*P*=0.039, paired *t*-test) in the presence of ivabradine, and GSIS was significantly (*P*=0.003, paired *t*-test) reduced. The significant basal secretion difference is indicated with a double-asterisk (**) and the significant GSIS differences are indicated with a single asterisk (*). (**f**) Fold-change representation of the GSIS values reported in **e**. (**g**) Basal human c-peptide secretion (percentage of total content) from hES derived β-like cells. Six such lines were analysed in three experiments each. Basal insulin secretion was significantly (*P*=0.01, *t*-test (equal variance)) increased by exposure to ivabradine. (**h**) GSIS of hES-derived β-like cells (*n*=6). No significant change (*P*=0.12, *t*-test (equal variance)) was found with ivabradine.

However, most of the genes identified here as differentially expressed in human β cell subsets currently lack a known role in this cell type. We therefore also wanted to ascertain whether functional differences between the subsets could be identified, particularly in regard to insulin secretion. Although FACS sorted single-cell suspensions of individual β cell subtypes lacked glucose responsiveness, GSIS was re-established by forming subtype aggregates in overnight co-culture (Supplementary Fig. 11). Post-assay viability was comparable (92–96%) in the different subtypes. Despite having equivalent insulin mRNA (Fig. 5a) and protein (Fig. 5b) content, differences in basal and GSIS were found. Basal insulin secretion in islet subtype aggregates was lowest for β1 (0.12±0.01 pmol per cell per h) and highest for β4 (0.25±0.23 pmol per cell per h; Fig. 5c), and the difference between these subtypes was significant (*P*=0.04). More importantly, there were clear differences in GSIS (Fig. 5c,d). Analyses of reaggregated subtypes from five normal islet samples revealed that the stimulation index of β1 aggregates (3.2±0.9 ×) was significantly higher than that of β2 (2.0±0.4 ×), β3 (1.9±0.6 ×) or β4 (1.4±0.2 ×). Given the clear differences in insulin secretion and the observation of differential expression of some genes involved in insulin secretion, we wondered whether differentially regulated genes of unknown function in human β cells may also be involved in this process. The HCN channel genes *HCN1* and *HCN4* were chosen for functional evaluation, because HCN2 was previously shown to be involved in insulin secretion in rats^23^,^24^. Ivabradine, a specific inhibitor of HCN channels^25^, was tested on whole human islets. Basal insulin secretion was significantly increased in the presence of the inhibitor (Fig. 5e), whereas GSIS was significantly suppressed (Fig. 5e,f). To prove that inhibition of HCN channels affected β cells directly and not via paracrine effects, ivabradine was tested on β-like cells generated from pluripotent precursors *in vitro*^26^. No other HCN-expressing cell types are present in this model. HCN inhibition significantly increased basal insulin secretion (Fig. 5g) as it had in intact islets. GSIS was not significantly altered (Fig. 5h). Together, these functional data show that the *HCN* genes differentially expressed in β cell subtypes play a role in modulating human insulin secretion, consistent with observations in rodents^27^. This ‘spot-test' of the differentially expressed gene list further strengthens our conclusion that human β cell subtypes differ in their insulin release kinetics.

### Diabetic islets have an abnormal β cell subtype distribution

Given that both gene expression analysis and insulin secretion analysis indicated functional heterogeneity among the β cell subtypes, we wondered whether disease states could affect the subset composition of human islets. To determine whether the alterations of islet structure and function associated with T2D^28^ affected subset distribution, islet samples from eight patients were examined. The total recoverable (live, non-doublet) β cell frequencies in these specimens fell within the range for normal specimens (15–45% of all endocrine cells), but the distribution of subtypes was highly variable between donors and clearly different from that of normal islets. Two distinct patterns were observed. In six out of eight patients (Fig. 1c–j), the frequencies of the ST8SIA1^+^ β3 and β4 populations (normally ∼18%) was unusually high. The differences in subtype percentages were highly significant between islets from normal individuals (both lean and obese) and those from T2D patients (Fig. 1l). In two additional patients, the β cell subset pattern was also very abnormal, but either the ST8SIA1^+^ population had disappeared (Fig. 1e) or the primary abnormality was a very high percentage of CD9^+^ cells (Fig. 1i). These data indicate that the frequencies of β cell subtypes are altered in the majority of individuals with T2D diabetes.

## Discussion

Most of the 200+ human cell types thought to exist were originally identified by nineteenth century histology methods. However, the advent of monoclonal antibody surface markers has led to the identification of important cellular subtypes among morphologically homogenous cell populations, most notably in the haematopoietic system^29^. Functional heterogeneity among β cells had long been suspected based on functional assays suggesting cells with different insulin release kinetics^2^,^9^, but until now it was impossible to prospectively isolate the subtypes. Here we demonstrate the existence of at least four distinct human β cell subtypes that can be separated based on cell surface marker expression. Clear differences in both basal and stimulated insulin secretion behaviour were observed between the subtypes, suggesting that the β subpopulations differ in their insulin release kinetics. These dissimilarities may serve to create smooth rather than sharp changes in insulin secretion in response to various stimuli and ensure that rapid shifts in blood glucose levels do not occur. Differentially expressed genes found here are good candidates to be modifiers of insulin release. We were able to demonstrate this directly for the HCN channels, which, until now, have not been known to play functional role in human β cells^30^. It seems unlikely that a single differentially expressed gene regulates the subtype insulin release kinetics. Instead, they probably act in concert. Future functional genomic studies should unravel the relative contributions of each factor. Recent work by Bader *et al.*^31^ shows that distinct β cell subtypes can also be found in the mouse. Using the Wnt pathway target gene *Fltp* as a marker, they demonstrated that these subtypes have distinct developmental origins, suggesting that this may also be the case with the human β cell subtypes described here. The genes differentially expressed in the murine subtypes only partially overlap with our human gene list, but like the human β cell subtypes, the murine subtypes differ in their insulin release kinetics. The clearly abnormal distribution of β cell subsets in the majority of our small T2D sample set raises the possibility that the subtypes differ in their susceptibility to metabolic stress^28^,^32^, proliferative capacity^33^ or differentiation state^34^. In normal islets, the ST8SIA1^+^ β3 and β4 populations, that is, those increased in most T2D patients, are less responsive to glucose. It is unclear at this time whether the four β cell subtypes are indicative of different developmental origins or whether they represent different functional and inter-convertible states of a single β cell type. However, given the remarkable reproducibility of their distribution percentages in normal islets obtained from many independent donors, it is unlikely that the subtypes could be the result of circadian, acute metabolic or ageing processes. Regardless of their ontogeny, the different properties of human β cell subtypes likely have an important impact on metabolic regulation and human disease processes.

## Methods

### Tissue sources and pancreatic cell isolation

Human pancreatic islets from normal and diabetic donors were obtained from the NIDDK-funded Integrated Islet Distribution Program at City of Hope^15^. These were collected from approved, consented cadaveric organ donors from which at least one other organ has been approved for transplantation and are exempt from human studies approval. Specimens were dispersed to single-cell suspensions by a 8–12 min digestion in 0.05% trypsin-EDTA (Thermo Fisher) at 37 °C with dispersal by a p1000 micropipetter every 3 min. Undispersed tissue was removed with a 40-μm cell strainer and dissociated cells were stored on ice in CMRL1066+2% FBS before antibody labelling. The number of samples analysed was determined primarily by material availability (especially in the case of T2D specimens) but was chosen to be sufficient for statistical analysis.

### Immunofluorescence imaging

Pancreatic cryosections (5 μm) from multiple donors were prepared from unfixed tissue in OCT blocks using a Reichert 2800 Frigocut (Reichert Scientific Instruments), treated with acetone for 10 min at −20 °C, and stored for up to 2 months at −80 °C. Non-specific labelling was blocked by pre-incubation in 2% goat serum (Hyclone) for 10 min. Initial labelling was performed with anti-human proinsulin (GS-9A8, Developmental Studies Hybridoma Bank) and detected/blocked with Cy3-conjugated monovalent Fab fragment goat anti-mouse IgG (Jackson ImmunoResearch 115-167-003) at high concentration (1:40 dilution) to permit detection of multiple primary antibodies of the same species. Subsequent labelling employed hybridoma supernatants diluted 1:20 (HPi2 or HPa3) and/or purified antibodies (anti-HCN1, ab84816, Abcam; biotinylated anti-CD9, 558749, BD Biosciences) diluted 1:200 in Dulbecco's phosphate buffered saline (DPBS) for 30 min and secondary labelling with 1:200 dilutions of Alexa488- or Alexa649-conjugated streptavidin, goat anti-mouse IgG(1,2a,2b) or goat anti-mouse IgM (Jackson ImmunoResearch, 115-545-164 and 115-166-075) as appropriate for 20 min. Nuclei were visualized with Hoechst 33342 (Molecular Probes). A Zeiss Axioskop 2 plus microscope (Carl Zeiss) was used for imaging.

### Flow cytometry and FACS

Dissociated cells were resuspended at 1 × 10^6^ cells per ml in CMRL1066+2% FBS before the addition of HIC3-2D12/HPa3 hybridoma supernatants (at a 1:20 dilution) and biotinylated anti-CD9 (eBioscience, 13-0098-80; 1:200 dilution) and incubation at 4 °C (for 30 min). After a wash with cold CMRL1066, cells were resuspended in CMRL1066+2% FBS containing a 1:200 dilution of PE-conjugated goat anti-mouse IgM (Jackson Immunoresearch) and a 1:100 dilution of PE-Cy7-conjugated streptavidin (BD Biosciences, 557598). After another wash, cells were resuspended in CMRL1066 (Corning)+5% mouse serum (Serotec) and held on ice (for 10 min) to block the secondary antibody. A final incubation with FITC-conjugated HIC0-3C5, APC-conjugated HIC1-2B4/HPi2, APC-Cy7-conjugated HIC0-3B3/HPx1, DHIC5-4D9/HPd3, anti-CD34 (BioLegend, 343514) and APC-H7-conjugated anti-CD45 (BD Biosciences, 560178) facilitated endocrine cell subfractionation and exclusion gating of acinar (HIC0-3B3/HPx1^+^), duct (DHIC5-4D9/HPd3^+^), haematopoietic (CD45^+^) and endothelial (CD34^+^) cells. The development and characterization of several of these antibodies have been previously described^12^. Cell doublets were excluded by pulse width measurement and propidium iodide staining was used to label dead cells for exclusion. Cells were analysed and sorted with a Cytopeia inFluxV-GS (Becton-Dickenson).

### β cell aggregation culture

A modified version of a co-aggregation culture developed for rat β cells^35^ was employed to permit functional assessment of FACS-sorted human β cells. On the day before the β cell isolation by FACS, a culture of mouse MS1 cells (American Type Culture Collection (ATCC) CRL-2279) was obtained from ATCC and established under standard adherent culture conditions. Following the isolation and brief storage of human β cells (at low glucose concentration), MS1 cells were recovered by trypsinization and combined with β cells at a 10:1 (MS1/β) ratio. This cell mixture was cultured in CMRL1066+5% human serum albumin+10 mM HEPES+antibiotic/antimycotic in a suspension (untreated) 24 w plate (Genesee Scientific) at 37 °C for 16 h. These cultures produced a mixture of single cells and small-to-medium sized aggregates (50–200 μm diameter), as shown in Supplementary Fig. 11. For basal and stimulated c-peptide measurement, cells and clusters were recovered, washed with Krebs-Ringer Bicarbonate (KRB)+2.8 mM glucose and assayed as described below.

### β-like cell differentiation and characterization

Human embryonic stem cell (hESC)-derived β-like cells were generated using our recently published differentiation approach with improvements at the last two stages^26^. For basal secretion and GSIS analysis, these hESC-derived spheres were transferred into tubes and washed twice with KRB+2.8 mM glucose, and assayed as described below.

### GSIS analysis

Samples (aggregated FACS-sorted β cells or hESC-derived spheres) were removed from media and equilibrated by incubation for 30 min at 37 °C in KRB+2.8 mM glucose with or without 30 mM ivabradine (Sigma-Aldrich). Cells were then transferred to fresh assay media (KRB+2.8 mM (basal) or 22.2 mM (stimulated) glucose with or without ivabradine for 1.5 h at 37 °C). After incubation, buffers were collected and frozen for subsequent human c-peptide-specific ELISA analysis (Mercodia). For hESC-derived spheres, total human c-peptide content analysis was performed by measurement of an aliquot of acidic ethanol lysed clusters by human c-peptide ELISA (Alpco). Statistical analyses of the results were performed using Graphpad Prism 4.0 (for analysis of variance tests) and Microsoft Excel (for mean, standard deviation and *t*-tests).

### RNA-seq library construction, sequencing and data analyses

Cell populations were sorted directly into Trizol LS (Invitrogen) and stored at −80 °C. For RNA recovery, samples were phenol/chloroform extracted before purification with RNeasy mini columns (Qiagen). Illumina RNA-Seq libraries were prepared from total RNA using the NuGen Ovation Pico kit to produce cDNA for 12 samples (four cell types from three donors). Fragmentation, blunt-ending, A-tailing and adapter ligation were then performed with the Illumina non-stranded RNA-Seq library prep kit to produced indexed libraries. The libraries were pooled and sequenced across two lanes of a hiSeq2000 to 100 bp single-read to an average depth of 19.7 million reads per sample. Gene expression values where generated for human genome release hg19 using RUM^36^. Differential expression was assessed using EdgeR^37^ using a two-factor analysis (donor id and β cell type). False discovery rates were calculated from *P*-values using the R function p.adjust in BH mode. For heat maps, reads per kb were normalized to the sample with the most reads. Normalization was done by a scaling the reads per kb values by the mean ratio for expressed genes, after removing the highly expressed genes. The median log_2_ expression for each gene was calculated for each donor then cell type-specific changes compared with this value. To reduce noise associated with differential contamination of the β cell subsets, genes known to be differentially expressed in non-β populations were excluded from the list of differentially expressed genes shown in Table 1, Fig. 4 and Supplementary Figs 6–9). Visualizations presented in Supplementary Figs 6–9 were generated using STRING (http://string-db.org/) (ref. 38).

### Antigen identification by cDNA expression library analysis

Rat C6 glioma cells (ATCC, CCL-107) were obtained from ATCC and transduced with a lentiviral library encoding version 5.1 of the human ORFeome library^39^ and screened by FACS for reactivity against HIC0-3C5. Two cycles of sorting HIC0-3C5^+^ C6 cells, amplification in culture and re-sorting yielded sufficient material for DNA and RNA recovery. PCR amplification of the cDNA insert from HIC0-3C5^+^ C6 cells was unsuccessful, so an RNAseq library was constructed and sequenced. The resulting sequences were searched for reads corresponding to ORFeome integrations and then compared with human cDNA sequences present in ORFeome 5.1 by BLAST^27^. Of the 17 mappable reads, 14 matched the 5′ region of ST8SIA1. In addition, the expression of ST8SIA1 was found to be consistently enriched in the RNA of FACS-sorted HIC0-3C5^+^ versus HIC0-3C5^−^ specimens by qRT–PCR and RNA-seq.

### Data availability

Gene expression data have been deposited in GEO (Gene Expression Omnibus) under accession code GSE80780. The authors declare that all other data supporting the findings of this study accompany the article or its Supplementary Information File.

## Additional information

**How to cite this article:** Dorrell, C. *et al.* Human islets contain four distinct subtypes of β cells. *Nat. Commun.* 7:11756 doi: 10.1038/ncomms11756 (2016).

## Supplementary Material

Supplementary Information Supplementary Figures 1-11

Peer review file

## Figures and Tables

**Table 1:** Genes significantly expressed in beta cells but with differential expression in ST8SIA1^+/−^ subtypes.

**Gene**		**FC (ST8SIA1^+^/ST8SIA1^−^)**	***P***	**FDR**
***ST8SIA1***	ST8 alpha-N-acetyl-neuraminide alpha-2,8-sialyltransferase 1	12.52	7.1E-11	0
*HCN1*	Hyperpolarization-activated cyclic nucleotide-gated potassium channel 1	3.03	1.5E-04	0.03
*DAB1*	Dab, reelin signal transducer, homologue 1 (*Drosophila*)	2.21	2.9E-05	0.01
*KCND3*	Potassium channel, voltage-gated Shal-related subfamily D, member 3	2.16	7.8E-09	0
*SIX3*	SIX homeobox 3	2.01	1.9E-09	0
*ATP8B1*	ATPase, aminophospholipid transporter, class I, type 8B, member 1	1.98	4.2E-04	0.05
*KCNJ8*	Potassium channel, inwardly rectifying subfamily J, member 8	1.9	6.8E-04	0.07
*SPP1*	Secreted phosphoprotein 1	1.87	4.7E-09	0
*TFF3*	Trefoil factor 3 (intestinal)	1.81	8.3E-13	0
*ITGA1*	Integrin, alpha 1	1.76	2.3E-04	0.04
*KCNE4*	Potassium channel, voltage-gated subfamily E regulatory beta subunit 4	1.76	6.4E-04	0.06
*RBM43*	RNA-binding motif protein 43	1.57	3.5E-04	0.04
*SCN3A*	Sodium channel, voltage-gated, type III, alpha subunit	1.57	7.3E-04	0.07
ABCC9/SUR2*	ATP-binding cassette, sub-family C (CFTR/MRP), member 9	1.54	3.2E-05	0.01
*SULF2*	Sulfatase 2	1.49	2.2E-05	0.01
*PON3*	Paraoxonase 3	1.43	5.3E-04	0.06
*GSN*	Gelsolin	1.4	7.3E-06	0
*OXCT1*	3-oxoacid CoA transferase 1	−1.33	2.8E-05	0.01
*TMCC3*	Transmembrane and coiled-coil domain family 3	−1.33	8.2E-05	0.02
NEUROD1*	Neuronal differentiation 1	−1.34	1.0E-03	0.09
*FAM159B*	Family with sequence similarity 159, member B	−1.36	6.7E-05	0.02
MAFB*	v-maf avian musculoaponeurotic fibrosarcoma oncogene homologue B	−1.38	1.4E-04	0.03
*SPTLC2*	Serine palmitoyltransferase, long-chain base subunit 2	−1.38	2.2E-04	0.03
*TNFRSF21*	Tumour necrosis factor receptor superfamily, member 21	−1.39	4.5E-07	0
*G6PC2*	Glucose-6-phosphatase 2	−1.46	3.2E-09	0
RFX6*	regulatory factor X, 6	−1.46	9.0E-07	0
*SCGB2A1*	Secretoglobin, family 2A, member 1	−1.49	8.9E-09	0
SLC2A2/GLUT2*	Solute carrier family 2 (facilitated glucose transporter), member 2	−1.53	7.2E-05	0.02
*HCN4*	Hyperpolarization-activated cyclic nucleotide-gated potassium channel 4	−1.77	9.4E-04	0.08

FC, fold change; FDR, false discovery rate.
