# Supplementary material for: Human islets contain four distinct subtypes of β cells
Source: Nat Commun. 2016 Jul 11;7:11756. doi: 10.1038/ncomms11756 (PMC4942571; doi:10.1038/ncomms11756)
Supplement: Supplementary Information — Supplementary Figures 1-11 [file ncomms11756-s1.pdf]

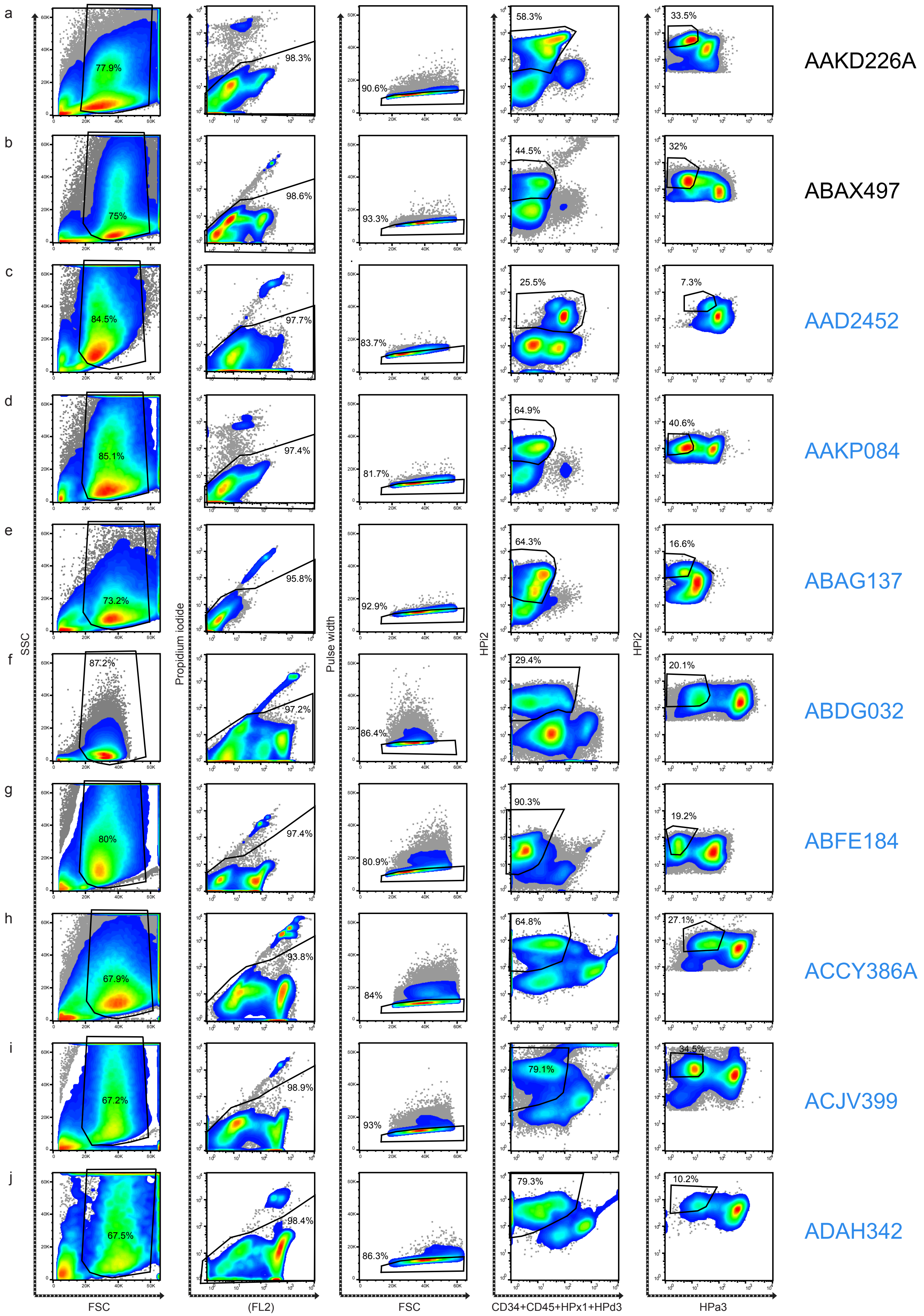

Supplementary Figure 1 – Isolation of β cells from healthy and T2D islets by FACS. Human islet samples were enzymatically dispersed and antibody labeled for flow cytometric analysis. For each islet specimen (a-j), sequential gating was used to exclude non-cell events (subpanel 1), dead cells (subpanel 2), doublets and larger cell clusters (subpanel 3), hematopoietic / endothelial / acinar / duct cells (subpanel 4), and to specifically select the β cell population (subpanel 5). SSC = Side Scatter; FSC = Forward Scatter; CD34, CD45; HPx1 (acinar), HPd3 (duct), HPa3 (α / δ / ε) and HPi2 (islet) are lineage-specific cell surface marking antibodies.

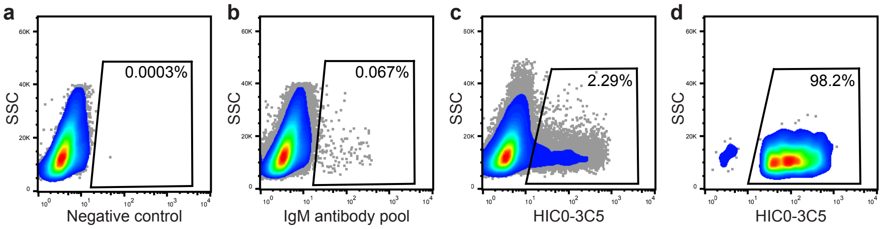

Supplementary Figure 2 – Flow cytometric screening of a cDNA library for antigen identification. C6 rat insulinoma cells were transduced with a human lentiviral cDNA library (ORFeome 5.1) and screened for reactivity against novel human monoclonal antibodies. (a,b) A pool of eight mouse IgM antibodies were exposed to library-containing C6 cells; a small fraction of cells (0.067%) were reactive. After expansion in cell culture, a 2.3% of these were found to be specifically reactive for HIC0-3C5 (c) and these were purified for insert sequencing (d).

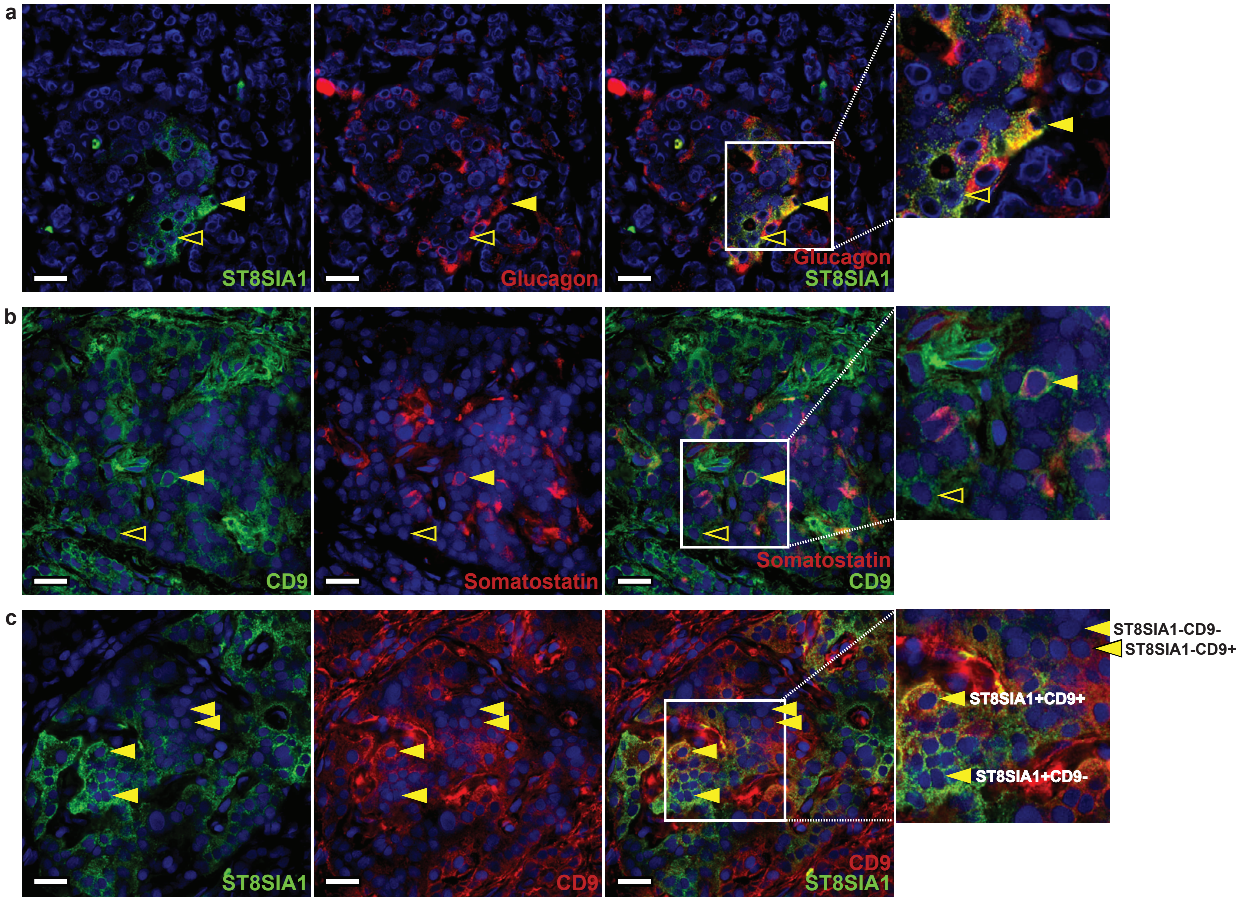

Supplementary Figure 3 – Subset marker expression on non-β endocrine cells. (a) Human pancreas co-labeled with antibodies recognizing ST8SIA1 and glucagon. ST8SIA1+ cells are mixture of glucagon+ alpha cells (solid arrows) and glucagon- β cells (hollow arrows). (b) Tissue co-labeled with antibodies recognizing CD9 and somatostatin. CD9+ cells are often somatostatin+ delta cells (solid arrows) but many are somatostatin- (hollow arrows). (c) Dual labeling of CD9 and ST8SIA1 reveals cells marked with neither, one, or both markers. Examples of each are indicated with arrows. Scale bar: 25 µm

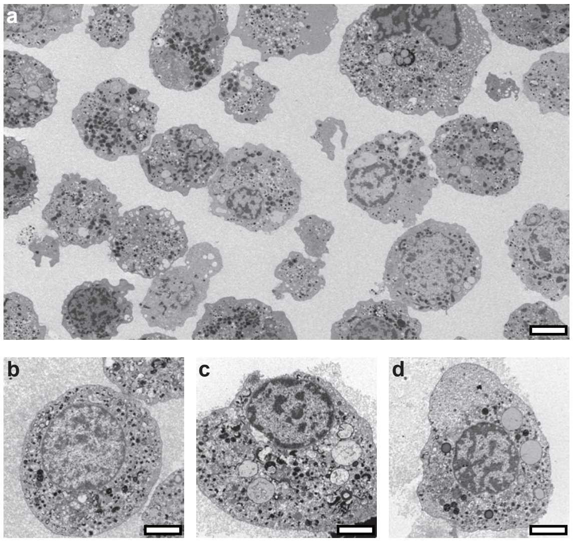

Supplementary Figure 4 – Ultrastructure of FACS-sorted human β cell populations reveal insulin granulation and comparable cell morphology. FACS-sorted β1 (a,b), β2 (c), and β3 (d) cells were fixed and stained en bloc with 1% uranyl acetate. Individual 60 nm sections were imaged by transmission electron microscopy and recorded with an Advanced Microscopy Techniques camera. Original magnifications: 890x (a) and 7100x (b-d). Scale bars: 15 μm (a) and 2 μm (b-d).

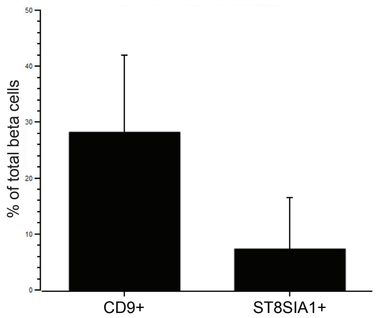

Supplementary Figure 5 – Quantification of β cell subset marker expression in pancreatic tissue. The graph illustrates the percentages of proinsulin+ islet cells expressing either CD9 or ST8SIA1 determined by visually scoring cells in a total of 100 tissue-resident islets. Tissue sections were prepared from cryopreserved blocks of two different human pancreata, with 50 islets scored from each.

a

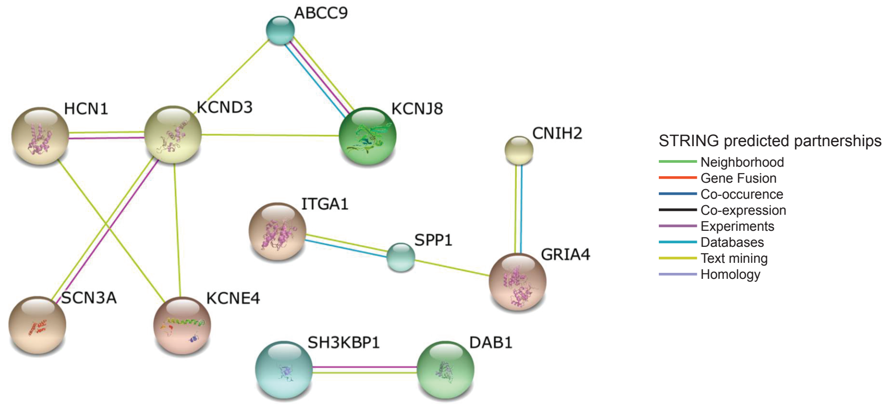

b Genes significantly enriched in ST8SIA1+ beta cells (vs. ST8SIA1-)

Gene		Transcript	FC (ST8SIA1+ / ST8SIA1-)	CPM	LR	p value	FDR
ST8SIA1	ST8 alpha-N-acetyl-neuraminide alpha-2,8-sialyltransferase 1	NM_003034	12.52	1.6	42.48	7.1E-11	0
ATP8A2	ATPase, Aminophospholipid Transporter, Class I, Type 8A, Member 2	NM_016529	3.92	1.9	14.28	1.6E-04	0.03
HCN1	Hyperpolarization activated cyclic nucleotide-gated potassium channel 1	NM_021072	3.03	2.4	14.38	1.5E-04	0.03
TSPAN18	Tetraspanin 18	NM_130783	2.8	2.7	13.89	1.9E-04	0.03
CHD5	Chromodomain Helicase DNA Binding Protein 5	NM_015557	2.6	2.7	10.53	1.2E-03	0.09
OPCML	Opioid Binding Protein/Cell Adhesion Molecule-Like	NM_001012393	2.45	7.4	22.25	2.4E-06	0
DAB1	Dab, Reelin Signal Transducer, Homolog 1 (Drosophila)	NM_021080	2.21	10.7	17.47	2.9E-05	0.01
SMOC1	SPARC Related Modular Calcium Binding 1	NM_022137	2.2	4.9	11.43	7.2E-04	0.07
KCND3	Potassium Channel, Voltage Gated Shal Related Subfamily D, Member 3	NM_004980	2.16	9.9	33.33	7.8E-09	0
PLCL1	Phospholipase C-Like 1	NM_006226	2.09	5.5	11.98	5.4E-04	0.06
TMEM159	Transmembrane Protein 159	NM_020422	2.07	6.7	11.71	6.2E-04	0.06
PCDH10	Protocadherin 10	NM_032961	2.06	6.0	16.29	5.4E-05	0.02
SIX3	SIX Homeobox 3	NM_005413	2.01	12.6	36.13	1.9E-09	0
ATP8B1	ATPase, Aminophospholipid Transporter, Class I, Type 8B, Member 1	NM_005603	1.98	7.3	12.43	4.2E-04	0.05
C1orf175/HEATR8	Chromosome 1 open reading frame 175	NM_001039464	1.94	4.9	14.64	1.3E-04	0.02
CORO2B	Coronin, Actin Binding Protein, 2B	NM_001190457	1.9	9.7	15.66	7.6E-05	0.02
KCNJ8	Potassium Channel, Inwardly Rectifying Subfamily J, Member 8	NM_004982	1.9	12.6	11.55	6.8E-04	0.07
SPP1	Secreted Phosphoprotein 1	NM_001040058	1.87	59.7	34.3	4.7E-09	0
GAP43	Growth Associated Protein 43	NM_001130064	1.84	15.9	10.48	1.2E-03	0.09
CDR2L	Cerebellar Degeneration-Related Protein 2-Like	NM_014603	1.83	4.6	11.1	8.7E-04	0.08
TFF3	Trefoil Factor 3 (Intestinal)	NM_003226	1.81	33.5	51.21	8.3E-13	0
MUC12	Mucin 12, Cell Surface Associated	NM_001164462	1.79	5.6	11.95	5.5E-04	0.06
OXR1	Oxidation Resistance 1	NM_018002	1.76	16.3	16.82	4.1E-05	0.01
ITGA1	Integrin, Alpha 1	NM_181501	1.76	11.7	13.54	2.3E-04	0.04
KIRREL3	Kin Of IRRE Like 3 (Drosophila)	NM_032531	1.76	7.7	13.2	2.8E-04	0.04
KCNE4	Potassium Channel, Voltage Gated Subfamily E Regulatory Beta Subunit 4	NM_080671	1.76	22.3	11.66	6.4E-04	0.06
TP53I11	Tumor Protein P53 Inducible Protein 11	NM_006034	1.75	16.4	33.3	7.9E-09	0
CREB3L1	CAMP Responsive Element Binding Protein 3-Like 1	NM_052854	1.71	5.2	10.62	1.1E-03	0.09
CNIH2	Cornichon Family AMPA Receptor Auxiliary Protein 2	NM_182553	1.63	13.2	18.89	1.4E-05	0.01
KIAA1644	KIAA1644	NM_001099294	1.59	77.7	27.74	1.4E-07	0
BCKDHA	Branched Chain Keto Acid Dehydrogenase E1, Alpha Polypeptide	NM_000709	1.58	9.3	10.22	1.4E-03	0.1
RBM43	RNA Binding Motif Protein 43	NM_198557	1.57	15.2	12.78	3.5E-04	0.04
SCN3A	Sodium Channel, Voltage Gated, Type III Alpha Subunit	NM_006922	1.57	14.5	11.4	7.3E-04	0.07
TMEM130	Transmembrane Protein 130	NM_001134450	1.55	19.2	11.92	5.6E-04	0.06
ABCC9	ATP-Binding Cassette, Sub-Family C (CFTR/MRP), Member 9	NM_020297	1.54	75.5	17.32	3.2E-05	0.01
GPX3	Glutathione Peroxidase 3	NM_002084	1.53	369.1	27.28	1.8E-07	0
MTUS1	Microtubule Associated Tumor Suppressor 1	NM_001001925	1.53	50.8	23.3	1.4E-06	0
ODZ4	odz, odd Oz/ten-m homolog 4 (Drosophila)	NM_001098816	1.52	16.8	14.7	1.3E-04	0.02
SULF2	Sulfatase 2	NM_198596	1.49	58.5	17.97	2.2E-05	0.01
SH3KBP1	SH3-Domain Kinase Binding Protein 1	NM_001024666	1.49	16.5	10.77	1.0E-03	0.09
DFNB31	Deafness, Autosomal Recessive 31	NM_001083885	1.44	18.3	17.11	3.5E-05	0.01
C13orf15	Chromosome 13 Open Reading Frame 15	NM_014059	1.44	99.5	13.19	2.8E-04	0.04
PON3	Paraoxonase 3	NM_000940	1.43	23.0	12	5.3E-04	0.06
MYOM1	Myomesin 1	NM_003803	1.42	32.7	12.24	4.7E-04	0.05
GSN	Gelsolin	NM_001127664	1.4	832.4	20.12	7.3E-06	0
NEFM	Neurofilament, Medium Polypeptide	NM_005382	1.4	130.7	19.67	9.2E-06	0
C1orf127	Chromosome 1 Open Reading Frame 127	NM_001170754	1.38	23.2	13.25	2.7E-04	0.04
PLD3	Phospholipase D Family, Member 3	NM_001031696	1.37	72.0	21.61	3.3E-06	0
NAGLU	N-Acetylglucosaminidase, Alpha	NM_000263	1.37	20.3	12.05	5.2E-04	0.06
GRIA4	Glutamate Receptor, Ionotropic, AMPA 4	NM_000829	1.37	42.2	11.58	6.7E-04	0.06
CITED2	Cbp/P300-Interacting Transactivator, With Glu/Asp-Rich Carboxy-Terminal Domain, 2	NM_006079	1.35	77.4	24.21	8.7E-07	0
PDK4	Pyruvate Dehydrogenase Kinase, Isozyme 4	NM_002612	1.33	138.1	18.16	2.0E-05	0.01
C17orf28	Chromosome 17 Open reading Frame 28	NM_030630	1.33	178.1	11.68	6.3E-04	0.06

Supplementary Figure 6 – Genes with significantly enriched expression in ST8SIA1+ β cells. Transcripts with significantly (FDR≤0.1, p≤0.001) elevated expression in ST8SIA1+ β cells compared to ST8SIA1- β cells and a fold change of at least 1.33x were identified. (a) Relationship networks between these genes predicted by STRING. (b) The list of 53 genes meeting these criteria; 11 genes were omitted due to low expression level or high probability of association with a contaminating population.

a

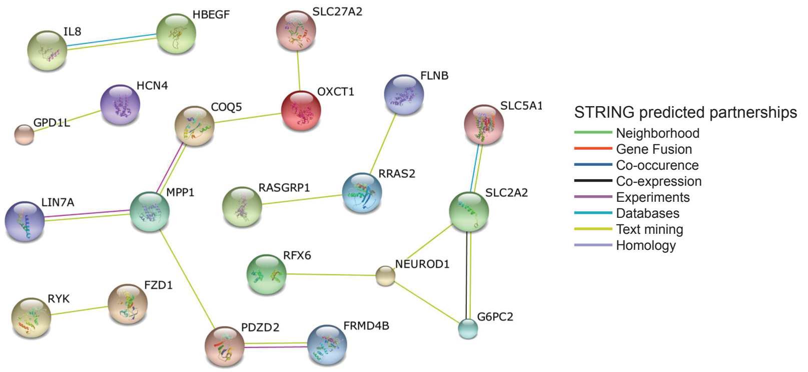

b Genes significantly enriched in beta ST8SIA1- cells (vs. ST8SIA1+)

Gene		Transcript	FC (ST8SIA1+ / ST8SIA1-)	CPM	LR	p value	FDR
FLNB	Filamin B, Beta	NM_001164317	-1.33	175.3	18.61	1.6E-05	0.01
OXCT1	3-Oxoacid CoA Transferase 1	NM_000436	-1.33	35.9	17.58	2.8E-05	0.01
TMCC3	Transmembrane And Coiled-Coil Domain Family 3	NM_020698	-1.33	59.4	15.52	8.2E-05	0.02
IL17RB	Interleukin 17 Receptor B	NM_018725	-1.33	65.8	13.2	2.8E-04	0.04
LRRFIP1	Leucine Rich Repeat (In FLII) Interacting Protein 1	NM_001137552	-1.34	106.0	15.09	1.0E-04	0.02
C12orf75	Chromosome 12 Open Reading Frame 75	NM_001145199	-1.34	60.3	11.88	5.7E-04	0.06
NEUROD1	Neuronal Differentiation 1	NM_002500	-1.34	74.0	10.81	1.0E-03	0.09
BNIP3	BCL2/Adenovirus E1B 19kDa Interacting Protein 3	NM_004052	-1.35	30.9	12.27	4.6E-04	0.05
DIAPH2	Diaphanous-Related Formin 2	NM_006729	-1.35	28.5	10.86	9.8E-04	0.08
PSD3	Pleckstrin And Sec7 Domain Containing 3	NM_015310	-1.36	146.9	18.93	1.4E-05	0.01
PDHB	Pyruvate Dehydrogenase (Lipoamide) Beta	NM_000925	-1.36	41.7	18.66	1.6E-05	0.01
MPP1	Membrane Protein, Palmitoylated 1, 55kDa	NM_001166462	-1.36	33.8	16.01	6.3E-05	0.02
FAM159B	Family With Sequence Similarity 159, Member B	NM_001164442	-1.36	44.6	15.9	6.7E-05	0.02
NVL	Nuclear VCP-Like	NM_206840	-1.37	23.8	12.09	5.1E-04	0.06
MAFB	V-Maf Avian Musculoaponeurotic Fibrosarcoma Oncogene Homolog B	NM_005461	-1.38	61.9	14.5	1.4E-04	0.03
SPTLC2	Serine Palmitoyltransferase, Long Chain Base Subunit 2	NM_004863	-1.38	45.3	13.64	2.2E-04	0.03
GPD1L	Glycerol-3-Phosphate Dehydrogenase 1-Like	NM_015141	-1.38	22.3	13.33	2.6E-04	0.04
TNFRSF21	Tumor Necrosis Factor Receptor Superfamily, Member 21	NM_014452	-1.39	470.6	25.48	4.5E-07	0
DNAJB4	DnaJ (Hsp40) Homolog, Subfamily B, Member 4	NM_007034	-1.39	13.6	10.23	1.4E-03	0.1
RASGRP1	RAS Guanyl Releasing Protein 1 (Calcium And DAG-Regulated)	NM_005739	-1.4	26.7	10.43	1.2E-03	0.1
VPS36	Vacuolar Protein Sorting 36 Homolog (S. Cerevisiae)	NM_016075	-1.41	24.3	11.89	5.6E-04	0.06
HBEGF	Heparin-Binding EGF-Like Grow th Factor	NM_001945	-1.42	22.8	14.87	1.2E-04	0.02
RYK	Receptor-Like Tyrosine Kinase	NM_001005861	-1.42	17.3	10.54	1.2E-03	0.09
RDH10	Retinol Dehydrogenase 10 (All-Trans)	NM_172037	-1.43	43.6	10.9	9.6E-04	0.08
PDZD2	PDZ Domain Containing 2	NM_178140	-1.44	33.9	15.77	7.1E-05	0.02
G6PC2	Glucose-6-Phosphatase, Catalytic, 2	NM_021176	-1.46	430.6	35.09	3.2E-09	0
RFX6	Regulatory Factor X, 6	NM_173560	-1.46	56.7	24.12	9.0E-07	0
GREM1	Gremlin 1, DAN Family BMP Antagonist	NM_001191323	-1.46	54.8	22.3	2.3E-06	0
DCBLD2	Discoidin, CUB And LCCL Domain Containing 2	NM_080927	-1.47	33.4	23.05	1.6E-06	0
ATP11A	ATPase, Class VI, Type 11A	NM_015205	-1.48	47.9	18.6	1.6E-05	0.01
RRAS2	Related RAS Viral (R-Ras) Oncogene Homolog 2	NM_001177315	-1.48	15.5	14.25	1.6E-04	0.03
RALGPS2	Ral GEF With PH Domain And SH3 Binding Motif 2	NM_152663	-1.48	13.8	13.24	2.7E-04	0.04
SCGB2A1	Secretoglobin, Family 2A, Member 1	NM_002407	-1.49	145.8	33.06	8.9E-09	0
C12orf76	Chromosome 12 Open Reading Frame 76	NM_207435	-1.52	20.9	14.7	1.3E-04	0.02
CASP2	Caspase 2, Apoptosis-Related Cysteine Peptidase	NM_032982	-1.52	28.5	14.7	1.3E-04	0.02
CBR4	Carbonyl Reductase 4	NM_032783	-1.52	11.2	10.33	1.3E-03	0.1
ZNF124	Zinc Finger Protein 124	NM_003431	-1.53	21.3	28.94	7.5E-08	0
PALM2	Paralemmin 2	NM_001037293	-1.53	18.5	15.8	7.0E-05	0.02
SLC2A2/GLUT2	Solute Carrier Family 2 (Facilitated Glucose Transporter), Member 2	NM_000340	-1.53	14.8	15.76	7.2E-05	0.02
MARK1	MAP/Microtubule Affinity-Regulating Kinase 1	NM_018650	-1.54	82.7	16.89	4.0E-05	0.01
CA5B	Carbonic Anhydrase VB, Mitochondrial	NM_007220	-1.55	24.7	12.02	5.3E-04	0.06
PANX1	Pannexin 1	NM_015368	-1.58	19.5	15.16	9.9E-05	0.02
MARS2	Methionyl-TRNA Synthetase 2, Mitochondrial	NM_138395	-1.6	8.6	12.69	3.7E-04	0.05
C6orf211	Chromosome 6 Open Reading Frame 211	NM_024573	-1.61	14.2	24.51	7.4E-07	0
MT1X	Metallothionein 1X	NM_005952	-1.62	120.5	16.25	5.6E-05	0.02
SLFN5	Schlafen Family Member 5	NM_144975	-1.62	15.1	11.16	8.4E-04	0.08
FRMD4B	FERM Domain Containing 4B	NM_015123	-1.63	7.5	11.74	6.1E-04	0.06
LNP1	Leukemia NUP98 Fusion Partner 1	NM_001085451	-1.63	6.1	10.38	1.3E-03	0.1
MT2A	Metallothionein 2A	NM_005953	-1.65	87.1	23.43	1.3E-06	0
PXK	PX Domain Containing Serine/Threonine Kinase	NM_017771	-1.65	19.3	15.86	6.8E-05	0.02
DCLRE1B	DNA Cross-Link Repair 1B	NM_022836	-1.65	7.9	10.67	1.1E-03	0.09
ZNF681	Zinc Finger Protein 681	NM_138286	-1.68	6.0	14.79	1.2E-04	0.02
HCN4	Hyperpolarization Activated Cyclic Nucleotide Gated Potassium Channel 4	NM_005477	-1.77	13.8	10.95	9.4E-04	0.08
AHNAK	AHNAK Nucleoprotein	NM_001620	-1.79	27.1	12.11	5.0E-04	0.06
TCEA3	Transcription Elongation Factor A (SII), 3	NM_003196	-1.85	8.5	11.42	7.3E-04	0.07
SLC27A2	Solute Carrier Family 27 (Fatty Acid Transporter), Member 2	NM_001159629	-1.86	5.3	16.64	4.5E-05	0.01
SLC39A11	Solute Carrier Family 39, Member 11	NM_001159770	-1.86	5.0	12.15	4.9E-04	0.06
PPARD	Peroxisome Proliferator-Activated Receptor Delta	NM_006238	-1.87	10.1	19.16	1.2E-05	0.01
COQ5	Coenzyme Q5 Homolog, Methyltransferase (S. Cerevisiae)	NM_032314	-1.89	9.4	21.33	3.9E-06	0
HS6ST2	Heparan Sulfate 6-O-Sulfotransferase 2	NM_001077188	-1.91	9.4	12.48	4.1E-04	0.05
DISP1	Dispatched Homolog 1 (Drosophila)	NM_032890	-1.96	14.0	13.27	2.7E-04	0.04
APCDD1L	Adenomatosis Polyposis Coli Dow n-Regulated 1-Like	NM_153360	-2.03	12.0	27.79	1.4E-07	0
NFIX	Nuclear Factor I/X (CCAAT-Binding Transcription Factor)	NM_002501	-2.04	12.8	40.47	2.0E-10	0
SLC5A1	Solute Carrier Family 5 (Sodium/Glucose Cotransporter), Member 1	NM_000343	-2.09	13.8	12.96	3.2E-04	0.04
MYCL1	L-myc-1 proto-oncogene protein	NM_001033081	-2.15	3.3	10.77	1.0E-03	0.09
AFAP1	Actin Filament Associated Protein 1	NM_001134647	-2.16	4.4	21	4.6E-06	0
DUSP19	Dual Specificity Phosphatase 19	NM_080876	-2.21	4.5	14.68	1.3E-04	0.02
FZD1	Frizzled Class Receptor 1	NM_003505	-2.25	9.1	23.87	1.0E-06	0
PPFIBP2	PTPRF Interacting Protein, Binding Protein 2 (Liprin Beta 2)	NM_003621	-2.33	6.3	17.67	2.6E-05	0.01
IL8	Interleukin 8	NM_000584	-2.38	33.4	15.97	6.4E-05	0.02
LIN7A	Lin-7 Homolog A (C. Elegans)	NM_004664	-3.27	1.9	10.46	1.2E-03	0.09
PCDH20	Protocadherin 20	NM_022843	-3.48	4.1	20.92	4.8E-06	0

Supplementary Figure 7 - Genes with significantly enriched expression in ST8SIA1- β cells. Transcripts with significantly (FDR≤0.1, p≤0.001) elevated expression in ST8SIA1- β cells compared to ST8SIA1+ β cells and a fold change of at least 1.33x were identified. (a) Relationship networks between these genes predicted by STRING. (b) The list of 71 genes meeting these criteria; 30 genes were omitted due to low expression level or high probability of association with a contaminating population.

a

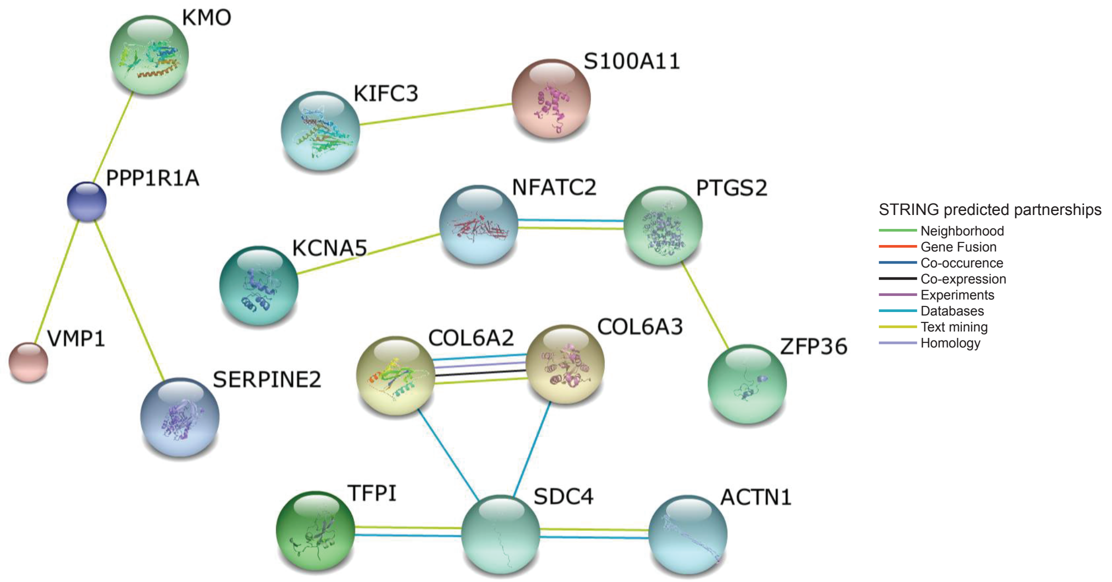

b

Genes significantly enriched in beta CD9+ cells (vs. CD9-)

Gene		Transcript	FC (CD9+ / CD9-)	CPM	LR	p value	FDR
LAPTM5	Lysosomal Protein Transmembrane 5	NM_006762	32.98	1.8	29.49	5.6E-08	0
IL1RN	Interleukin 1 Receptor Antagonist	NM_173843	28.65	1.3	18	2.2E-05	0.01
CD9	CD9 Molecule	NM_001769	9.12	29.2	163.9	1.6E-37	0
PRDM1	PR Domain Containing 1, With ZNF Domain	NM_001198	7.53	1.9	17.81	2.4E-05	0.01
TACSTD2	Tumor-Associated Calcium Signal Transducer 2	NM_002353	6.15	2.2	11.78	6.0E-04	0.08
COL6A3	Collagen, Type VI, Alpha 3	NM_057167	5.97	1.2	10.77	1.0E-03	0.1
COL6A2	Collagen, Type VI, Alpha 2	NM_058175	4.76	-1.1	11.95	5.5E-04	0.07
SLC44A4	Solute Carrier Family 44, Member 4	NM_001178044	3.59	2.0	16.57	4.7E-05	0.01
CD74	CD74 Molecule, Major Histocompatibility Complex, Class II Invariant Chain	NM_001025159	2.81	3.8	13.8	2.0E-04	0.04
BMP2	Bone Morphogenetic Protein 2	NM_001200	2.56	4.0	11.31	7.7E-04	0.09
PMEPA1	Prostate Transmembrane Protein, Androgen Induced 1	NM_199169	2.26	3.9	15.13	1.0E-04	0.02
TSPAN33	Tetraspanin 33	NM_178562	2.21	11.5	11.98	5.4E-04	0.07
KMO	Kynurenine 3-Monooxygenase (Kynurenine 3-Hydroxylase)	NM_003679	2.12	3.3	12.64	3.8E-04	0.06
MIA2	Melanoma Inhibitory Activity 2	NM_054024	2.12	22.8	11.34	7.6E-04	0.09
TFPI	Tissue Factor Pathw ay Inhibitor (Lipoprotein-Associated Coagulation Inhibitor)	NM_006287	2.11	4.7	12.27	4.6E-04	0.07
NPC1L1	NPC1-Like 1	NM_001101648	2.1	40.5	32.8	1.0E-08	0
SYNM	Synemin, Intermediate Filament Protein	NM_145728	2.1	12.9	14.27	1.6E-04	0.03
SDC4	Syndecan 4	NM_002999	2.06	15.1	22.43	2.2E-06	0
KCNA5	Potassium Channel, Voltage Gated Shaker Related Subfamily A, Member 5	NM_002234	2.04	9.9	18.78	1.5E-05	0.01
RBP4	Retinol Binding Protein 4, Plasma	NM_006744	1.93	139.4	16.62	4.6E-05	0.01
TMED6	Transmembrane Emp24 Protein Transport Domain Containing 6	NM_144676	1.92	34.8	17.42	3.0E-05	0.01
KIFC3	Kinesin Family Member C3	NM_005550	1.9	10.7	11.14	8.4E-04	0.09
PTGS2/COX2	Prostaglandin-Endoperoxide Synthase 2 (Prostaglandin G/H Synthase And Cyclooxygenase)	NM_000963	1.88	159.1	36.15	1.8E-09	0
SERPINE2	Serpin Peptidase Inhibitor, Clade E (Nexin, Plasminogen Activator Inhibitor Type 1), Member 2	NM_001136528	1.87	22.0	12.5	4.1E-04	0.06
ACTN1	Actinin, Alpha 1	NM_001130004	1.75	19.5	17.67	2.6E-05	0.01
RIPK4	Receptor-Interacting Serine-Threonine Kinase 4	NM_020639	1.75	44.4	15.08	1.0E-04	0.02
PPP1R1A	Protein Phosphatase 1, Regulatory (Inhibitor) Subunit 1A	NM_006741	1.74	192.8	18.76	1.5E-05	0.01
TTC9	Tetratricopeptide Repeat Domain 9	NM_015351	1.74	7.8	11.04	8.9E-04	0.09
AHNAK	AHNAK Nucleoprotein	NM_001620	1.74	27.1	10.64	1.1E-03	0.1
RAI14	Retinoic Acid Induced 14	NM_001145525	1.69	32.1	20.17	7.1E-06	0
MCOLN3	Mucolipin 3	NM_018298	1.68	17.8	11.93	5.5E-04	0.07
GNAL	Guanine Nucleotide Binding Protein (G Protein), Alpha Activating Activity Polypeptide, Olfactory Type	NM_002071	1.67	14.2	14.95	1.1E-04	0.02
AK5	Adenylate Kinase 5	NM_012093	1.62	7.7	11.4	7.3E-04	0.08
SEL1L3	Sel-1 Suppressor Of Lin-12-Like 3 (C. Elegans)	NM_015187	1.61	7.8	13.52	2.4E-04	0.04
VWA5B2	Von Willebrand Factor A Domain Containing 5B2	NM_138345	1.59	18.5	11.87	5.7E-04	0.07
ZFP36	ZFP36 Ring Finger Protein	NM_003407	1.56	25.7	27.73	1.4E-07	0
S100A11	S100 Calcium Binding Protein A11	NM_005620	1.52	17.1	11.72	6.2E-04	0.08
NFATC2	Nuclear Factor Of Activated T-Cells, Cytoplasmic, Calcineurin-Dependent 2	NM_001136021	1.49	23.9	12.7	3.7E-04	0.06
CREB5	CAMP Responsive Element Binding Protein 5	NM_004904	1.4	160.1	25.29	4.9E-07	0
SLC7A14	Solute Carrier Family 7, Member 14	NM_020949	1.4	69.5	11.36	7.5E-04	0.09
EPB41L1	Erythrocyte Membrane Protein Band 4.1-Like 1	NM_012156	1.39	64.2	12.24	4.7E-04	0.07
HMGCS1	3-Hydroxy-3-Methylglutaryl-CoA Synthase 1 (Soluble)	NM_002130	1.38	76.9	20.16	7.1E-06	0
IER3	Immediate Early Response 3	NM_003897	1.38	165.1	19.06	1.3E-05	0.01
PGM2L1	Phosphoglucomutase 2-Like 1	NM_173582	1.38	47.5	11.15	8.4E-04	0.09
AKAP13	A Kinase (PRKA) Anchor Protein 13	NM_007200	1.36	124.3	22.04	2.7E-06	0
VMP1	Vacuole Membrane Protein 1	NM_030938	1.34	108.3	22.39	2.2E-06	0

Supplementary Figure 8 - Genes with significantly enriched expression in CD9+ β cells. Transcripts with significantly (FDR≤0.1, p≤0.001) elevated expression in CD9+ β cells compared to CD9- β cells and a fold change of at least 1.33x were identified. (a) Relationship networks between these genes predicted by STRING. (b) The list of 47 genes meeting these criteria; 23 genes were omitted due to low expression level or high probability of association with a contaminating population.

a

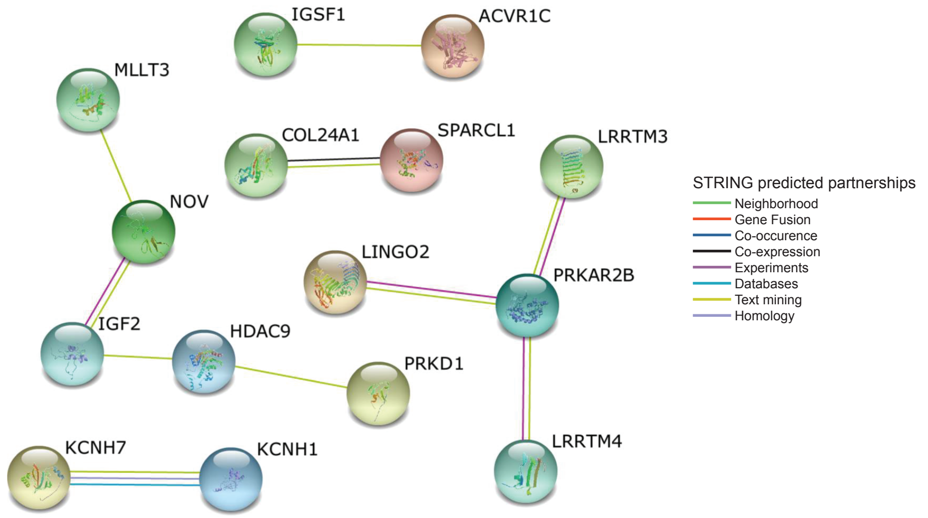

b Genes significantly enriched in CD9- beta cells (vs. CD9+)

Gene		Transcript	FC (CD9+ / CD9-)	CPM	LR	p value	FDR
SATB1	SATB Homeobox 1	NM_002971	-1.33	60.4	20.69	5.4E-06	0
KCNMB2	Potassium Channel Subfamily M Regulatory Beta Subunit 2	NM_005832	-1.33	81.0	18.66	1.6E-05	0.01
MBNL3	Muscleblind-Like Splicing Regulator 3	NM_133486	-1.34	77.8	25.26	5.0E-07	0
SOX5	SRY (Sex Determining Region Y)-Box 5	NM_006940	-1.34	23.9	12.78	3.5E-04	0.05
PTPN13	Protein Tyrosine Phosphatase, Non-Receptor Type 13 (APO-1/CD95 (Fas)-Associated Phosphatase)	NM_080683	-1.35	70.8	19.27	1.1E-05	0
FKBP5	FK506 Binding Protein 5	NM_001145775	-1.35	153.5	17.52	2.9E-05	0.01
HDAC9	Histone Deacetylase 9	NM_014707	-1.35	112.2	16.24	5.6E-05	0.02
LSAMP	Limbic System-Associated Membrane Protein	NM_002338	-1.35	86.4	14.44	1.5E-04	0.03
PCDH7	Protocadherin 7	NM_032457	-1.35	67.5	12.08	5.1E-04	0.07
IGF2	Insulin-Like Grow th Factor 2	NM_001007139	-1.35	112.0	11.7	6.3E-04	0.08
CPEB2	Cytoplasmic Polyadenylation Element Binding Protein 2	NM_001177382	-1.36	42.2	11.16	8.4E-04	0.09
NOV	Nephroblastoma Overexpressed	NM_002514	-1.37	35.0	13.82	2.0E-04	0.04
METAP1	Methionyl Aminopeptidase 1	NM_015143	-1.38	27.9	13.22	2.8E-04	0.05
ARL4C	ADP-Ribosylation Factor-Like 4C	NM_005737	-1.38	109.4	13.17	2.9E-04	0.05
CDK8	Cyclin-Dependent Kinase 8	NM_001260	-1.41	29.5	15	1.1E-04	0.02
CHL1	Cell Adhesion Molecule L1-Like	NM_006614	-1.42	54.7	16.49	4.9E-05	0.01
IGSF1	Immunoglobulin Superfamily, Member 1	NM_001170961	-1.42	24.7	15.78	7.1E-05	0.02
MLLT3	Myeloid/Lymphoid Or Mixed-Lineage Leukemia (Trithorax Homolog, Drosophila); Translocated To, 3	NM_004529	-1.42	17.8	11.02	9.0E-04	0.09
SLC8A1	Solute Carrier Family 8 (Sodium/Calcium Exchanger), Member 1	NM_021097	-1.43	156.8	19.58	9.7E-06	0
PLK2	Polo-Like Kinase 2	NM_006622	-1.44	42.2	16.68	4.4E-05	0.01
SEMA6A	Sema Domain, Transmembrane Domain (TM), And Cytoplasmic Domain, (Semaphorin) 6A	NM_020796	-1.46	133.8	23.25	1.4E-06	0
CNKSR2	Connector Enhancer Of Kinase Suppressor Of Ras 2	NM_014927	-1.49	31.3	16.34	5.3E-05	0.01
ACVR1C	Activin A Receptor, Type IC	NM_001111031	-1.5	39.6	12.14	4.9E-04	0.07
MAF1	MAF1 Homolog (S. Cerevisiae)	NM_032272	-1.51	19.6	10.63	1.1E-03	0.1
ASXL2	Additional Sex Combs Like Transcriptional Regulator 2	NM_018263	-1.53	42.7	21.29	4.0E-06	0
LINGO2	Leucine Rich Repeat And Ig Domain Containing 2	NM_152570	-1.53	24.7	10.65	1.1E-03	0.1
SIM1	Single-Minded Family BHLH Transcription Factor 1	NM_005068	-1.54	13.8	17.33	3.1E-05	0.01
TAGLN3	Transgelin 3	NM_013259	-1.54	42.4	13.51	2.4E-04	0.04
PRKAR2B	Protein Kinase, CAMP-Dependent, Regulatory, Type II, Beta	NM_002736	-1.55	24.2	20.35	6.5E-06	0
ASB9	Ankyrin Repeat And SOCS Box Containing 9	NM_001168531	-1.55	44.7	16.15	5.9E-05	0.02
WASF1	WAS Protein Family, Member 1	NM_003931	-1.55	19.9	14.81	1.2E-04	0.03
TSPAN1	Tetraspanin 1	NM_005727	-1.56	27.4	24.51	7.4E-07	0
AMPH	Amphiphysin	NM_001635	-1.56	32.9	11.63	6.5E-04	0.08
LRRTM3	Leucine Rich Repeat Transmembrane Neuronal 3	NM_178011	-1.58	48.7	25.24	5.1E-07	0
SPATA18	Spermatogenesis Associated 18	NM_145263	-1.58	17.8	11.96	5.4E-04	0.07
NAV3	Neuron Navigator 3	NM_014903	-1.59	18.3	18.42	1.8E-05	0.01
KCNH7	Potassium Channel, Voltage Gated Eag Related Subfamily H, Member 7	NM_033272	-1.6	21.6	20.12	7.3E-06	0
KCNH1	Potassium Channel, Voltage Gated Eag Related Subfamily H, Member 1	NM_002238	-1.6	21.7	12.87	3.3E-04	0.05
ADAMTS5	ADAM Metallopeptidase With Thrombospondin Type 1 Motif, 5	NM_007038	-1.65	16.1	14.56	1.4E-04	0.03
RALYL	RALY RNA Binding Protein-Like	NM_173848	-1.68	8.9	10.88	9.7E-04	0.1
MDFIC	MyoD Family Inhibitor Domain Containing	NM_199072	-1.73	20.6	23.37	1.3E-06	0
SPARCL1	SPARC-Like 1 (Hevin)	NM_004684	-1.77	10.9	11.18	8.3E-04	0.09
STARD9	StAR-Related Lipid Transfer (START) Domain Containing 9	NM_020759	-1.84	9.6	12.05	5.2E-04	0.07
DGKB	Diacylglycerol Kinase, Beta 90kDa	NM_004080	-1.99	27.8	22.66	1.9E-06	0
STMN2	Stathmin 2	NM_001199214	-2.02	132.4	44.29	2.8E-11	0
KLHL1	Kelch-Like Family Member 1	NM_020866	-2.1	6.3	11.2	8.2E-04	0.09
CLVS1	Clavesin 1	NM_173519	-2.14	4.9	18.21	2.0E-05	0.01
CNTN5	Contactin 5	NM_014361	-2.18	14.5	18.76	1.5E-05	0.01
LRRTM4	Leucine Rich Repeat Transmembrane Neuronal 4	NM_024993	-2.24	10.5	36.3	1.7E-09	0
THSD7A	Thrombospondin, Type I, Domain Containing 7A	NM_015204	-2.3	58.2	25.5	4.4E-07	0
NELL2	NEL-Like 2 (Chicken)	NM_001145108	-2.52	7.2	15.09	1.0E-04	0.02
CDH18	Cadherin 18, Type 2	NM_001167667	-2.56	28.6	21.66	3.3E-06	0
GRIP1	Glutamate Receptor Interacting Protein 1	NM_001178074	-2.98	5.3	20.44	6.2E-06	0
NPY	Neuropeptide Y	NM_000905	-3.08	212.3	36.49	1.5E-09	0
CCDC81	Coiled-Coil Domain Containing 81	NM_001156474	-3.17	2.5	14.37	1.5E-04	0.03
B3GALT1	UDP-Gal:BetaGlcNAc Beta 1,3-Galactosyltransferase, Polypeptide 1	NM_020981	-3.2	2.3	13.11	2.9E-04	0.05
ITM2A	Integral Membrane Protein 2A	NM_001171581	-3.47	3.4	14.73	1.2E-04	0.03
PRKD1	Protein Kinase D1	NM_002742	-3.53	4.8	11.25	8.0E-04	0.09
ZNF804A	Zinc Finger Protein 804A	NM_194250	-3.6	1.8	11.23	8.1E-04	0.09
PTPRD	Protein Tyrosine Phosphatase, Receptor Type, D	NM_002839	-3.75	17.9	54.25	1.8E-13	0
COL24A1	Collagen, Type XXIV, Alpha 1	NM_152890	-4.39	2.4	26.43	2.7E-07	0

Supplementary Figure 9 – Genes with significantly enriched expression in CD9- β cells. Transcripts with significantly (FDR≤0.1, p≤0.001) elevated expression in CD9- β cells compared to CD9+ β cells and a fold change of at least 1.33x were identified. (a) Relationship networks between these genes predicted by STRING. (b) The list of 60 genes meeting these criteria; 15 genes were omitted due to low expression level or high probability of association with a contaminating population.

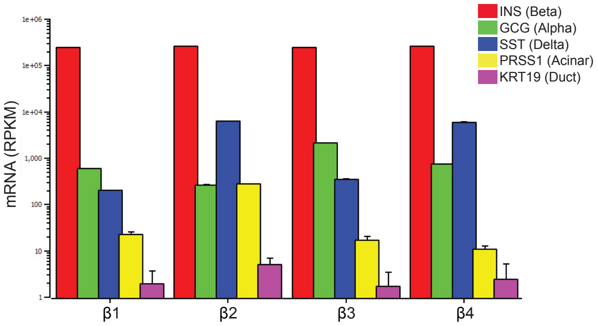

Supplementary Figure 10 – Analysis of β cell population purity. Normalized RNA-seq values (Reads Per Kilobase per Million; RPKM) for genes associated with cell-type specific gene expression are indicated. Exocrine cell contamination, indicated by PRSS1 and KRT19 expression, was <0.1% in each population. As indicated by detection of somatostatin and glucagon tags, the principal contaminants in each population were either delta cells (2.4% in β2, 2.3% in β4) or alpha cells (0.2% in β1, 0.8% in β3).

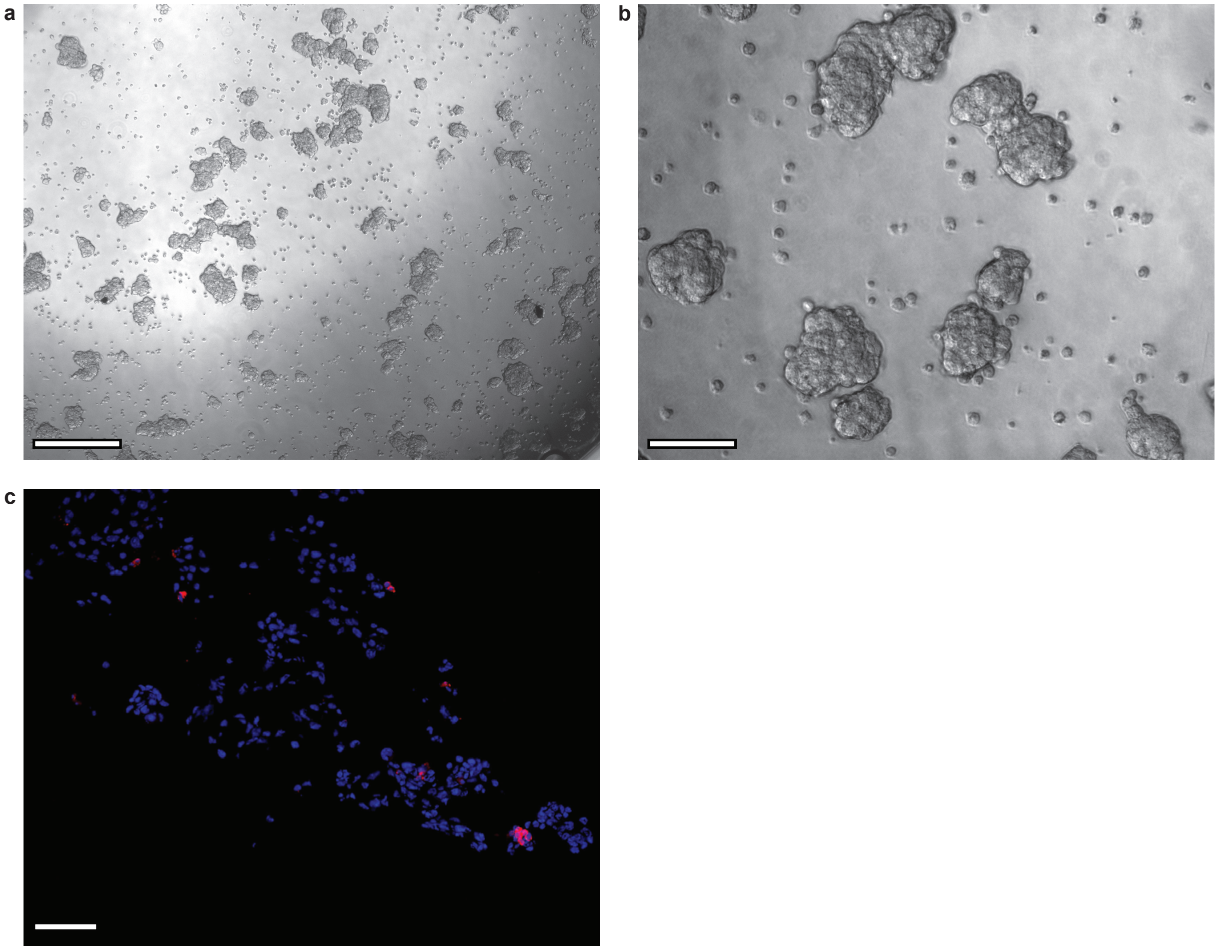

Supplementary Figure 11 – Reaggregation of FACS-sorted β cells to restore function. Overnight suspension culture of β cells with mouse endothelial cell line MS1 at a 1:10 ratio produces spheroid aggregates (a) 40x magnification of bright field image of aggregates in culture (b) 100x magnification; (c) Proinsulin positive cells are associated with or incorporated in clusters of negative (MS1) cells (100x magnification, 5μm cryosections, nuclei visualized with Hoechst 33342). Scale bars: 500 μm (a), 100 μm (b,c).
