# Supplementary material for: Human islets contain four distinct subtypes of β cells
Source: Nat Commun. 2016 Jul 11;7:11756. doi: 10.1038/ncomms11756 (PMC4942571; doi:10.1038/ncomms11756)
Supplement: Peer review file [file ncomms11756-s2.pdf]

Editorial Note: This manuscript has been previously reviewed at another journal that is not operating a transparent peer review scheme. It was accepted without further rounds of review at Nature Communications
